# Necessity for a National Trauma Center

**DOI:** 10.5812/traumamon.18109

**Published:** 2014-08-01

**Authors:** Ali Ebrahimi

**Affiliations:** 1 Trauma Research Center, Baqiyatallah University of Medical Sciences, Tehran, IR Iran

**Keywords:** Trauma Centers, Disasters, Violence, Injuries

Trauma places a massive burden on national economies, costing countries billions of dollars each year in healthcare, law enforcement and productivity. According to annual reports by police and government officials, road traffic accidents are the second most common cause of mortality after cardiovascular events among civilians in Iran (1). Immediate trauma assistance can reduce the impact of the traumatic event, expedite the return of normal function and reduce the likelihood of post-traumatic psychosocial problems. When an accident, workplace injury, disaster or traumatic death occurs, the victims experience overwhelming emotions including shock, confusion, pain, sorrow, anguish and grief. As traumatic situations can occur at any time or place, trauma centers (TC) must have the capacity to provide management for all trauma victims, on a 24-hour basis. These centers must respond rapidly in mass casualty incidents and disasters. TC are also expected to provide fellowships to teach trainees the various components of triage and emergency care, as well as run organized trauma hospitals (2). TC must enhance preparedness through teaching, training and practice, while providing ready response teams equipped to rescue and resuscitate victims of various disasters, and then to evacuate and transfer them to other centers across our country.

One of the problems with trauma care in our country is a lack of coordination among nationwide TC and trauma system organizations. Although clinical management of trauma patients has improved vastly in recent decades, however, decreasing the burden of injuries necessitates an organized approach in order to prevent and manage trauma in the context of an organized national trauma system (3). Training, education, research and treatment of trauma victims are key priorities for the national trauma center (NTC) which requires a significant investment of resources in order to ensure trauma and disaster training for all clinicians across the country. The NTC must also focus on providing clinical and academic leadership with regard to disasters and trauma care. Thus, we feel it prudent to seek a national service provider to coordinate trauma and crisis management. 
